# Expression of ribosomal S6 kinase 4 in bladder cancer and its correlation with clinicopathological features

**DOI:** 10.3389/fonc.2026.1757293

**Published:** 2026-02-20

**Authors:** Zhenzhen Li, Gongchen Wang, Siyu Tan, Yingmei Wang, Junyi Feng, CHen Wei, Jia Chai, Yanru Yang, Zhiyong Yin, Xiaoyan Zhou, Jing Ma, Linni Fan

**Affiliations:** 1 Yan’an Medical College, Yan’an University, Yan’an, China; 2 State Key Laboratory of Holistic Integrative Management of Gastrointestinal Cancers, Department of Pathology, School of Basic Medicine and Xijing Hospital, Fourth Military Medical University, Xi’an, China; 3 Department of Cardiology, The First Affiliated Hospital of Air Force Medical University, Xi’an, China

**Keywords:** bladder cancer, immunohistochemistry, prognosis, RSK4, tissue microarray

## Abstract

**Context:**

Bladder cancer management is challenged by limited therapeutic targets and heterogeneous treatment responses. Ribosomal S6 kinase 4 (RSK4) has been established as an oncogenic driver in several malignancies, although its clinical significance in bladder cancer remains undefined.

**Objective:**

To evaluate RSK4 protein expression in bladder cancer specimens and assess its association with clinicopathologic features and patient outcomes.

**Design:**

RSK4 expression was analyzed by immunohistochemistry in a retrospective cohort of 143 bladder cancer specimens, including 93 cases represented in a tissue microarray. Statistical analyses were performed to evaluate the associations between RSK4 expression levels, standard clinicopathological parameters, and overall survival.

**Results:**

RSK4 immunoreactivity was detected in 65.7% (94/143) of tumor tissue samples but in 36.5% (23/63) of matched normal urothelial tissue samples (P < 0.0001). Elevated RSK4 expression was significantly correlated with the following established markers of disease progression: muscularis propria invasion (P < 0.001), high tumor grade (P < 0.01), advanced TNM stage (P < 0.001), lymph node metastasis (P < 0.01), and distant metastasis (P < 0.05). No significant associations were observed with patient age, sex, or tumor size. Multivariate analysis confirmed RSK4 as an independent predictor of reduced overall survival (HR = 2.34, 95% CI 1.42–3.85, P < 0.001). Subcellular studies indicate that RSK4 overexpression enhances the invasive and metastatic capabilities of bladder cancer cell lines, and vice versa.

**Conclusions:**

This study elucidates the expression pattern and mechanism of action of RSK4 in bladder urothelial carcinoma, confirming that its overexpression is a key factor for predicting poor prognosis. This discovery highlights the potential value of RSK4 as a significant therapeutic target, providing a new theoretical basis for improving clinical outcomes in bladder cancer.

## Introduction

Bladder cancer (BLCA) ranks among the most common malignant tumors of the urinary system, with more than 570,000 new cases and approximately 210,000 deaths reported globally each year, indicating a rising incidence trend (1). Owing to the lack of specific clinical manifestations in the early stages, most patients are diagnosed at the muscle-invasive stage (muscle-invasive bladder cancer, MIBC). Currently, the response rate to immunotherapy for MIBC remains limited, and intervenable molecular targets are scarce, making treatment options inadequate. Consequently, identifying novel biomarkers for prognostic assessment and targeted therapy in bladder cancer has become an urgent priority in the field of bladder cancer research.

Ribosomal S6 protein kinase 4 (RSK4) is a member of the p90 ribosomal S6 kinase (RSK) family. As a serine/threonine protein kinase, it functions downstream of the MAPK signaling pathway and participates in the regulation of various biological processes, including cell proliferation, differentiation, survival, and migration (2). Recent studies have revealed that RSK4 has diverse functions across different tumor types, acting as either an oncogene or a tumor suppressor with high tissue specificity. Research has indicated that in ovarian cancer (3), glioma (4), and clear cell renal carcinoma (5), RSK4 accelerates tumor progression by activating prosurvival signaling axes such as the PI3K/AKT, mTOR, or MAPK signaling pathways, thereby promoting cell cycle progression and inhibiting apoptosis. However, studies in endometrial cancer (6) and breast cancer (7) have yielded opposite conclusions, suggesting that RSK4 exerts tumor-suppressing effects through the dual mechanisms of epigenetic regulation and inhibition of metastasis. Specifically, after restoring expression via promoter demethylation, RSK4 functions as an antimetastatic gene by suppressing invasion, enhancing adhesion, and downregulating proliferation signals.

Our group’s prior research demonstrated that RSK4 expression is significantly elevated in esophageal squamous cell carcinoma (ESCC) (8). Its expression level is independently and positively correlated with local recurrence after radiotherapy and reduced overall survival, making it a viable prognostic biomarker. Both *in vivo* and *in vitro* experiments consistently confirmed that gene silencing (shRNA) or pharmacological inhibition (BI-D1870) of RSK4 can block DNA damage repair, induce apoptosis and mitotic catastrophe, increase tumor radiosensitivity by 1.5–2.3-fold, and significantly prolong progression-free survival in mouse xenograft models. These findings establish RSK4 as a core molecule driving ESCC radioresistance and a precise radiotherapy target for intervention. Furthermore, RSK4 is highly expressed in renal clear cell carcinoma (9), and its level is significantly correlated with high WHO/ISUP grading and poor prognosis. *In vitro* overexpression experiments further confirmed that RSK4 significantly enhances cancer cell proliferation and clonogenic capacity, suggesting that it plays a role as an oncogene in the malignant progression of renal clear cell carcinoma. The research team has filed patents for RSK4 small-molecule inhibitors, including APY29 and CZC-54252, which efficiently inhibit RSK4 enzymatic activity, significantly promote ESCC apoptosis, and effectively block proliferation, invasion, and migration. These findings lay a solid foundation for subsequent clinical translation. Although RSK4 has demonstrated potential clinical value in urological tumors such as renal cell carcinoma, research on its role in bladder cancer remains unexplored. Therefore, this study employed immunohistochemical staining using the EnVision method to detect RSK4 expression in bladder cancer tissues. The correlations between its expression levels and patients’ clinical pathological characteristics (such as tumor stage, muscle layer invasion, and tumor grade) and prognosis were analyzed. Furthermore, the potential mechanism of RSK4 was explored in BLCA cell lines to verify its potential as a new therapeutic target for bladder cancer treatment.

## Materials and methods

### Clinical pathological data

This study included 143 consecutive patients with bladder cancer who underwent surgical resection or transurethral resection of bladder tumors at the First Affiliated Hospital of Air Force Medical University from 2020–2024. TMAs were constructed from 93 of these cases. Among the 143 patients, 110 were male and 33 were female, with a male-to-female ratio of 3.3:1. The mean patient age was 67.7 years (range: 35–86 years), and the mean tumor size was approximately 4.14 cm (range: 0.4–16.0 cm). All included cases were re-evaluated and graded according to the WHO Classification of Tumors of the Urinary System (5th edition, 2022). Based on tumor depth of invasion, bladder cancer can be categorized primarily into muscle-invasive bladder cancer (MIBC) and nonmuscle-invasive bladder cancer (NMIBC). Among these, 64 patients had MIBC, and 79 had NMIBC. By stage, 47 cases were T1–2, and 56 cases were T3–4. Based on histological morphology, 27 patients had low-grade urothelial carcinoma, and 116 patients had high-grade urothelial carcinoma. The different histological subtypes included 130 cases of pure urothelial carcinoma (UC-Pure), 4 cases of urothelial carcinoma with squamous differentiation (UC-SD), and 4 cases of urothelial carcinoma with glandular differentiation (UC-GD). Thirteen patients had lymph node involvement and distant metastasis (LNI and DM), and 130 patients had no LNI or DM. All the specimens were reviewed by two pathologists to ensure diagnostic accuracy. Concurrently, 113 patients with BLCA underwent follow-up, yielding survival data. The survival time from surgery to the follow-up cutoff was calculated and averaged to be 26.5 months (range: 1–82 months). Specimen collection and experimental procedures received approval from the Ethics Committee of Xijing Hospital.

### Immunohistochemistry

RSK4 expression in BLCA was detected using the EnVision two-step immunohistochemical method. The staining procedure followed that of reference (10), and the specific steps were as follows: 4-mm-thick paraffin-embedded sections and tissue microarrays (TMAs) were subjected to routine dewaxing in water, followed by three rinses with PBS. Antigen retrieval for RSK4 was performed using an alkaline buffer (pH=9.0, Tris-EDTA). A 3% H_2_O_2_-methanol solution was subsequently added for 20 minutes to inactivate endogenous peroxidase activity. The sections were then incubated with 5% (m/v) bovine serum albumin (BSA) for 30 minutes to block nonspecific antibody-binding sites. RSK4 (rabbit anti-human monoclonal antibody, clone EP1982Y, Abcam, UK) diluted 1:75 was added, and the samples were incubated overnight at 4 °C. The next day, the samples were heated to 37 °C for 1 hour and then washed three times with PBS, after which the EnVision secondary antibody (Dako, Denmark) was added, and the samples were incubated at room temperature for 40 minutes. After three PBS washes, the sections were developed with DAB, counterstained with hematoxylin, dehydrated, and mounted. Positive results appear as yellow–brown granules, with RSK4 positively expressed in the cytoplasm.

### Evaluation of immunohistochemical staining

Semiquantitative analysis of the staining results was performed according to reference (11). Each slide was scored based on the proportion of positive cells and the intensity of cellular staining. The staining intensity was graded as follows: no staining (unstained) = 0, light brown = 1, moderate brown = 2, and dark brown = 3. The corresponding percentage areas were denoted as a0, a1, a2, and a3, respectively. The H score calculation formula was as follows: H score = 1 × a1 + 2 × a2 + 3 × a3, with a total score range of 0–300. In this study, the median H score of 140 was used as the cutoff value: an H score ≥ 140 was defined as positive expression, and an H score < 140 was defined as negative expression.

### Cell lines, plasmids, and transfection

The human bladder carcinoma (BLCA) cell lines T24 and J82 and TCCSUP were purchased from Wuhan Punoise Biotechnology Co., Ltd., and were cultured at 37 °C in RPMI 1640 medium (HyClone, Waltham, MA, USA) supplemented with 20% fetal bovine serum (FBS, Gibco, Carlsbad, CA, USA) and maintained at 5% CO_2_. In this study, the pcDNA3.1/Neo-RSK4 plasmid was used to stably transfect a bladder cancer (BLCA) cell line to overexpress the human RSK4 protein. Transfection was performed using Lipofectamine 2000 transfection reagent (InGen, Carlsbad, CA, USA) strictly following the manufacturer’s protocol. The specific procedure was as follows: After the cells were seeded in a 6-well plate and allowed to reach 90% confluence, 4.0 mg of plasmid DNA was added to each well and mixed with 10 µl of Lipofectamine 2000 for transfection. After 48 hours, the cells were digested with trypsin and transferred to 10 cm culture dishes containing 300 mg/mL G418 (Gibco). Clones were isolated using single-cell cloning techniques and subsequently expanded. The protein expression levels of RSK4 were detected via Western blot analysis.

### Lentivirus-based shRNA transduction

This study employed lentiviral transduction technology to achieve stable low expression of the human RSK4 gene in a bladder cancer (BLCA) cell line. A lentivirus carrying a shRNA sequence targeting RSK4 (shRSK4) was constructed and provided by G-Kai Gene Technology Co., Ltd. (China). To increase transduction efficiency, cells were subjected to two consecutive viral infections, followed by selection for stable clones using pyrimethamine (1 mg/mL, Merck KGaA, Germany). The specific procedure was as follows: One day prior to infection, the cells were seeded at a density of 1×10^5^ cells/mL in 6-well plates (1 mL per well) and cultured overnight until they reached confluence. For transduction, each well received 1 mL of enhanced infection medium, 20 µL of RSK4 lentivirus stock, and 5 µL of polybrene (at a final concentration of 5 µg/mL), with the multiplicity of infection (MOI) set at 10. After 12 h of infection, the cell medium was replaced with complete medium for continued culture. Green fluorescent protein (GFP) expression was assessed by fluorescence microscopy at 24, 48, and 72 h to evaluate transduction efficiency. When the cell confluence reached 60–70%, 1 mg/mL puromycin was added for continuous selection to obtain stable cells with low RSK4 expression.

### Western blot

Cell lysis was performed in buffer (1–5 mg/ml pepitin, 1–5 mg/ml leucinopeptidase, 2 mmol/l Na_3_VO_4_, 1 mmol/l sodium fluoride, 10 mmol/l glycerophosphate, 1 mmol/l benzenesulfonyl fluoride, 0.1% Tween-20, 1 mmol/l DTT, 1 mmol/l EDTA, 150 nmol/l NaCl, and 50 mmol/l Tris-HCl). Protein quantification was performed using a BCA protein assay (Pierce, Thermo). Protein samples (15 mg per lane) were separated by 10% SDS–PAGE, transferred to PVDF membranes (Millipore, Billerica, MA, USA), and visualized using chemiluminescence (Pierce, Thermo). Immunoblotting was performed using a rabbit anti-human RSK4 antibody (Abcam, UK, 1:500 dilution).

### Matrigel invasion assay

The cells were resuspended in serum-free 1640 medium at a density of 5 × 10^5^ cells/mL. Next, 100 µL of cell suspension was added to the upper chamber of the Transwell (precoated with 1 mg/mL Matrigel matrix). Next, 600 µL of 1640 medium containing 30% fetal bovine serum was added to the lower chamber as the chemotactic stimulus. The culture plate was incubated at 37 °C in a 5% CO_2_ incubator for 24 hours. After incubation, the chamber was removed, and the inner surface of the upper chamber membrane was gently wiped with a cotton swab to remove nonmigrated cells. Cells that migrated to the lower surface of the membrane were fixed and stained with 0.1% crystal violet solution for subsequent counting and analysis. All experiments were independently repeated at least three times.

### Statistics

SPSS software (version 27.0, SPSS Inc., Chicago, IL, USA) was used for data analysis. The chi-square test was used to evaluate differences and correlations between RSK4 expression levels and clinical pathological characteristics in patients with bladder cancer (BLCA). Univariate and multivariate survival analyses were conducted separately. Survival curves were constructed using the Kaplan–Meier method, and curve differences were compared via the log-rank test. Cox regression models were used to analyze survival risk factors and differences in BLCA. All experiments were repeated three times. Cellular experimental data are presented as the mean ± standard deviation. Intergroup comparisons were performed using one-way analysis of variance (ANOVA), with all tests being two-tailed. A P value < 0.05 was considered to indicate statistical significance.

## Results

### RSK4 expression was significantly increased in bladder carcinoma compared with normal urothelium

**Figure 1:**
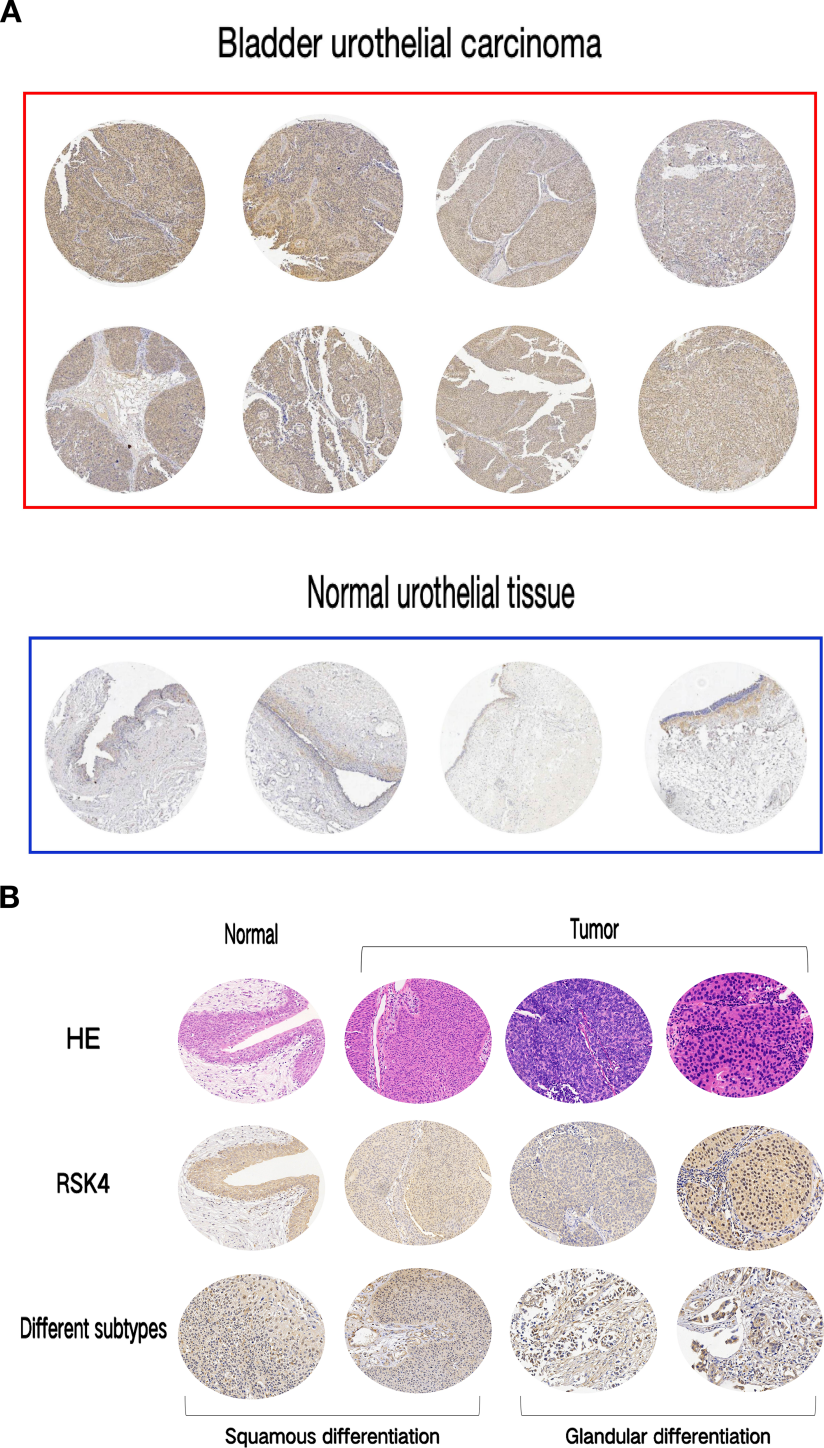
Expression of RSK4 in bladder urothelial carcinoma and normal urothelial tissues. **(A)** Tissue microarray images of immunohistochemical staining for RSK4 in bladder urothelial carcinoma and normal urothelium. **(B)** Hematoxylin and eosin (HE) and immunohistochemical staining images of RSK4 in bladder urothelial carcinoma and normal urothelium and immunohistochemical staining in different subtypes (SD, GD) (100×). **(C)** Expression levels of RSK4 in bladder urothelial carcinoma and normal urothelium. t test, ****P < 0.0001.

**Figure 2:**
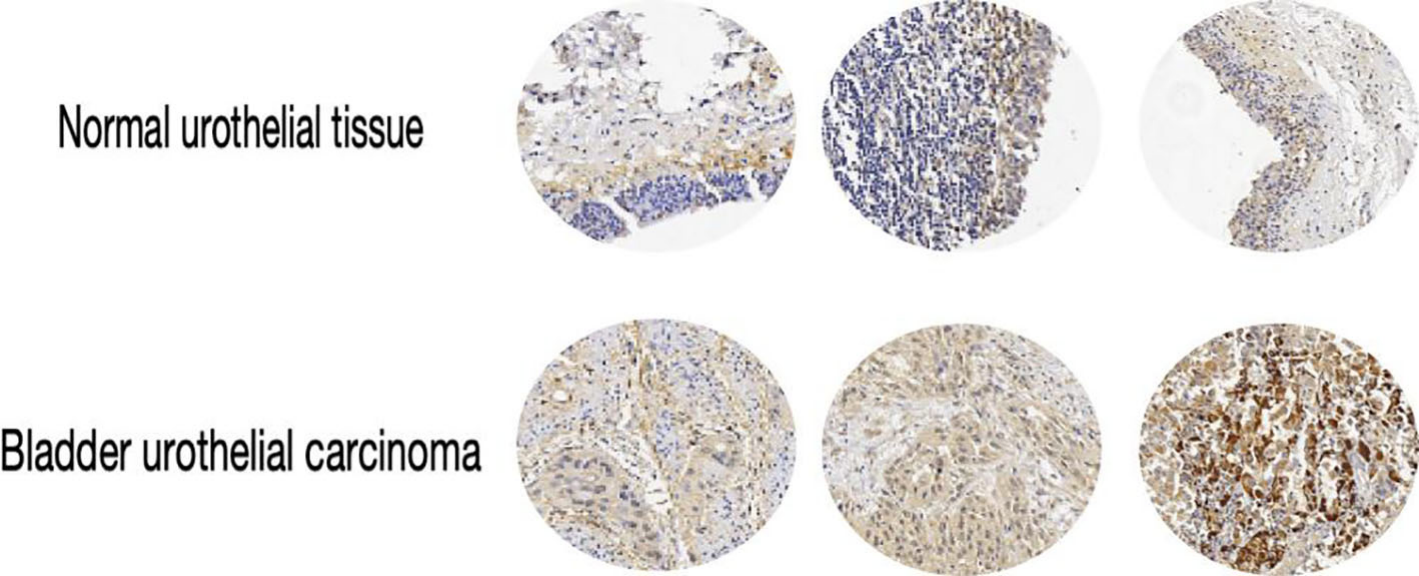
Representative images of RSK4 immunohistochemical staining in three cases of bladder urothelial carcinoma and their paired normal tissues (100×). Above each case is the adjacent normal urothelium; below is the corresponding neoplastic tissue; significantly increased RSK4 expression is observed in the neoplastic areas.

RSK4 is overexpressed in bladder urothelial carcinoma (BLCA), with distinct expression levels among different histological subtypes. The positive expression rate of RSK4 in BLCA tissues was 65.7% (94/143), whereas it was 36.5% (23/63) in adjacent normal urothelial tissues. Chi-square analysis revealed that RSK4 was significantly overexpressed in BLCA tissue compared with normal tissue (*P* < 0.0001) (**Figures 1A–C**; **Table 1**). The RSK4 expression rates in the UC-Pure, UC-SD, and UC-GD subtypes were 60% (84/130), 75% (3/4), and 100% (4/4), respectively (**Figure 1B**). Additionally, in 3 patients, RSK4 expression levels were significantly higher in bladder urothelial carcinoma tissue than in the corresponding normal urothelium (**Figure 2**).

**Table 1:** Expression of RSK4 in bladder urothelial carcinoma and normal urothelium.

Tissue	rsk4-	rsk4+	Pos.rate	n	χ^2^	P
Bladder urothelial carcinoma	49	94	65.7%	143	15.22	<0.0001
Normal urothelial tissue	40	23	36.5%	63		

### RSK4 expression was significantly correlated with tumor invasion depth, histologic grade, and pT stage

**Figure 3:**
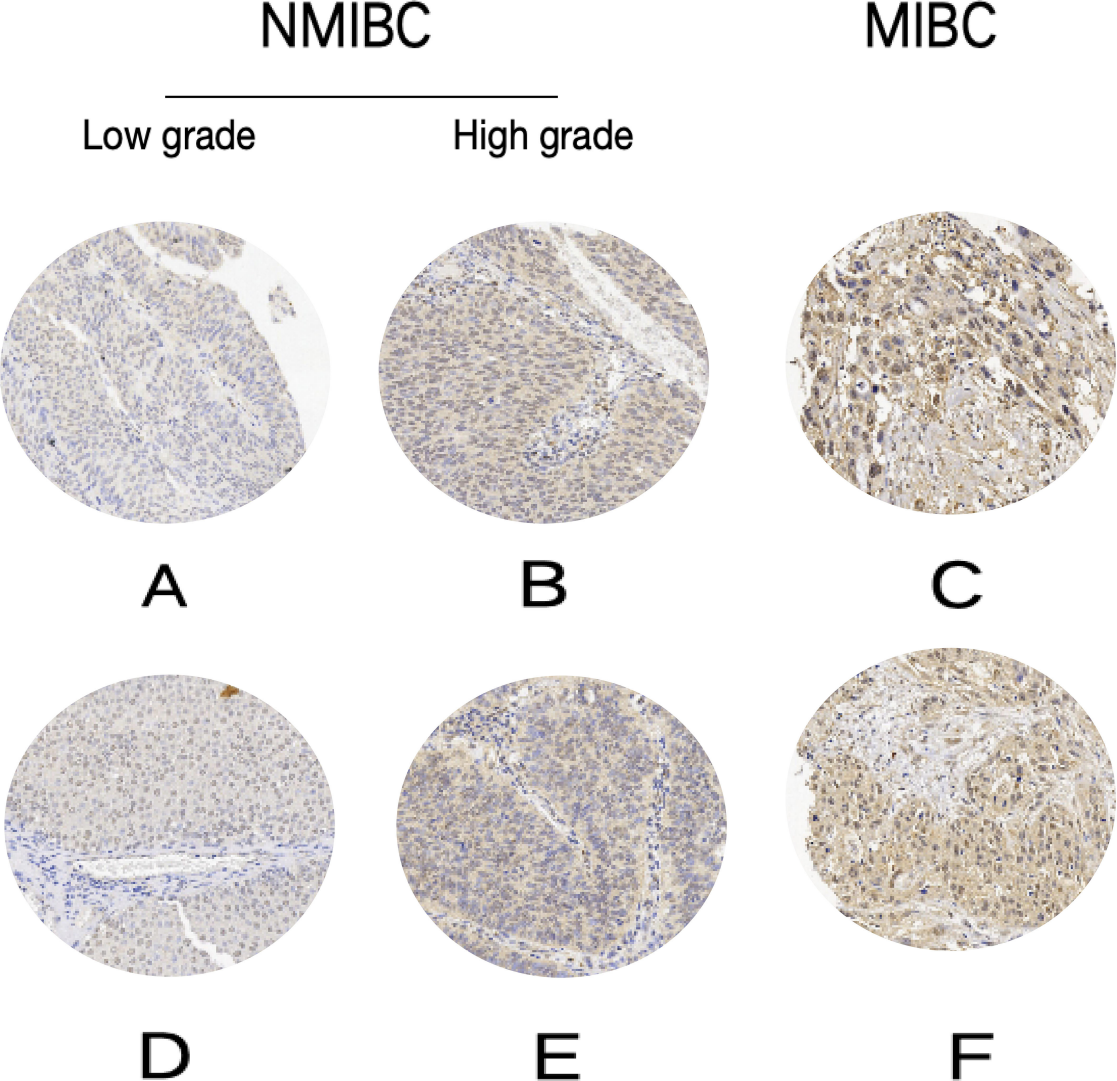
Immunohistochemical RSK4 expression patterns across NMIBC grades and MIBC stages (100×). **(A, D)** Low-grade NMIBC. **(B, E)** High-grade NMIBC. **(C, F)** MIBC.

RSK4 expression was present in 89.1% (57/64) of the patients with MIBC and in 46.8% (37/79) of the patients with NMIBC. RSK4 expression levels increased with increasing depth of invasion (**Figures 3A–C, F**). Similarly, RSK4 was expressed in 82 (82/116, 70.7%) high-grade tumors and 12 (12/27, 44.4%) low-grade tumors, as well as in 30 (30/47, 63.8%) pT1–2 stage tumors and 48 (48/56, 85.7%) pT3–4 stage tumors. RSK4 expression increased with increasing histologic grade (**Figures 3D, E**). RSK4 expression was significantly correlated with tumor invasion depth (*P* < 0.0001), histologic grade (*P* = 0.0132), and pT stage (*P* = 0.0119) (*χ²* test; **Table 2**). Furthermore, RSK4 expression correlated with lymph node involvement and distant metastasis (*P* = 0.0354, **Table 2**). However, age, sex, tumor size, and histological subtype were not significantly associated with RSK4 expression (*P*>0.05, **Table 2**).

**Table 2:** Cross-analysis of RSK4 immunostaining with clinicopathological features in all patients with BLCA.

Clinical pathological features (n)	RSK4+	RSK4-	P-value
Age(years)			>0.9999
≥60(116)	77(66.4%)	39(33.6%)	
<60(26)	17(65.4%)	9(34.6%)	
Gender			0.1450
Male(110)	76(69.1%)	34(30.9%)	
Female(33)	18(54.5%)	15(45.5%)	
Tumor size(cm)			0.5921
≥4(69)	47(68.1%)	22(31.9%)	
<4(68)	43(63.2%)	25(36.8%)	
Histological type			0.5088
UC,pure(130)	84(64.6%)	46(35.4%)	
UC-SD(4)	3(75%)	1(25%)	
UC-GD(4)	4(100%)	–	
Histologic Grade (WHO2022)			0.0132
High Grade(116)	82(70.7%)	34(29.3%)	
Low Grade(27)	12(44.4%)	15(55.6%)	
Invasion depth			<0.0001
MIBC(64)	57(89.1%)	7(10.9%)	
NMIBC(79)	37(46.8%)	42(53.2%)	
pTstage			0.0119
рТ1-pT2(47)	30(63.8%)	17(36.2%)	
рТ3-рТ4(56)	48(85.7%)	8(14.3%)	
LNI and DM			0.0354
yes(13)	12(92.3%)	1(7.7%)	
no(130)	82(63.1%)	48(36.9%)	

RSK4 expression predicts poor prognosis in patients with BLCA.

### RSK4 expression predicts poor prognosis in patients with BLCA

**Figure 4:**
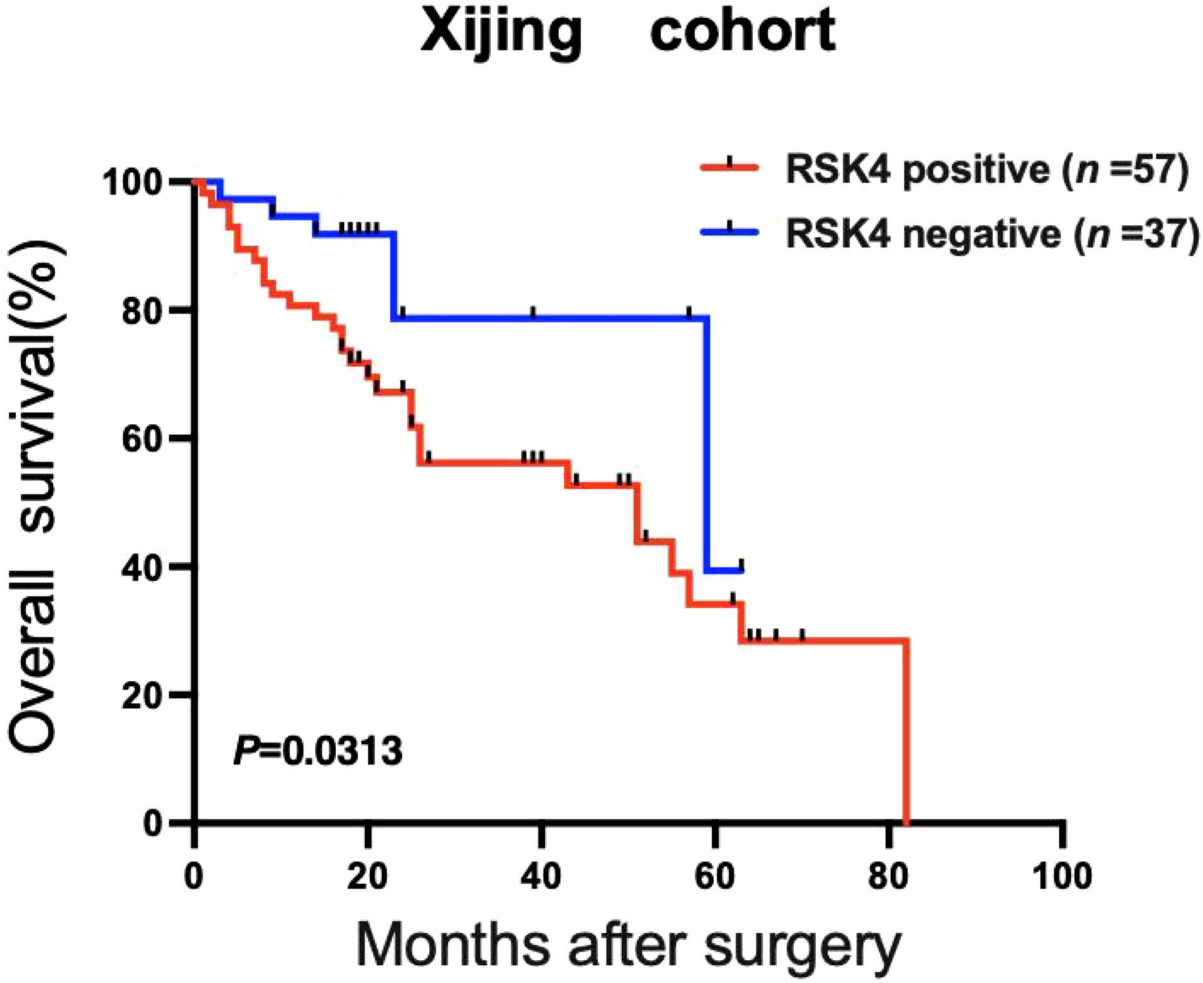
Positive RSK4 expression predicts poor prognosis in patients with BLCA. Kaplan–Meier survival curves demonstrated a positive correlation between RSK4 expression status and prognosis in 94 patients with bladder cancer. A log-rank test revealed that positive RSK4 expression was associated with poor prognosis (*P* = 0.0313).

In addition to patients with missing data, 94 patients were followed up for survival. Among them, 55 tumors were RSK4 positive, and 34 were RSK4 negative. The median survival period for patients with RSK4-positive tumors was 25 months (range: 1–82 months). Log-rank analysis revealed significantly lower survival rates in RSK4-positive patients than in RSK4-negative patients (*P* = 0.0313, **Table 3**). RSK4 expression is a poor prognostic indicator in patients with bladder cancer (**Figure 4**). Univariate analysis revealed that high RSK4 expression (*P* = 0.0313), high pT stage (*P* < 0.0001), high grade (*P* = 0.0461), lymph node involvement, and distant metastasis (*P* = 0.0032) were associated with poor survival rates (**Table 3**). However, RSK4 expression was not significantly different according to multivariate analysis (**Table 3**).

**Table 3:** Univariate and multivariate analysis of survival in 94 BLCA cases with follow-up information.

Variable	Mean survival in month (95%CI)	Univariate P-value	HR (95%CI)	Multivariate P-value
Age(years)		0.3637	1.000	0.986
≥60(n=76)	27(23–31)			
<60(n=17)	23(15–31)			
Gender		0.9777	0.529	0.351
Male(n=73)	27(23–32)			
Female(n=21)	22(16–28)			
Histological type		/	0.647	0.459
UC,pure(n=87)	25(22–30)			
UC-SD(n=3)	52(8–97)			
UC-GD(n=2)	40			
Tumor size(cm)		0.3735	0.514	0.156
≥4(n=43)	30(23–37)			
<4(n=45)	21(17–25)			
RSK4		0.0313	1.791	0.389
Positive(n=57)	29(24–35)			
Negative(n=37)	22(17–26)			
Invasion depth		0.2634	1.061	0.928
MIBC(n=38)	30(22–38)			
NMIBC(n=56)	24(20–27)			
Histologic Grade		0.0461	0.484	0.418
High Grade(n=74)	26(22–30)			
Low Grade(n=20)	25(19–32)			
pTstage		<0.0001	6.160	0.001
pT1-pT2(n=33)	32(25–39)			
pT3-pT4(n=33)	25(17–32)			
LNI and DM		0.0032	1.235	0.765
yes (n=9)	16(8–25)			
no (n=85)	27(23–31)			

### RSK4 silencing and overexpression cell models were established

**Figure 5:**
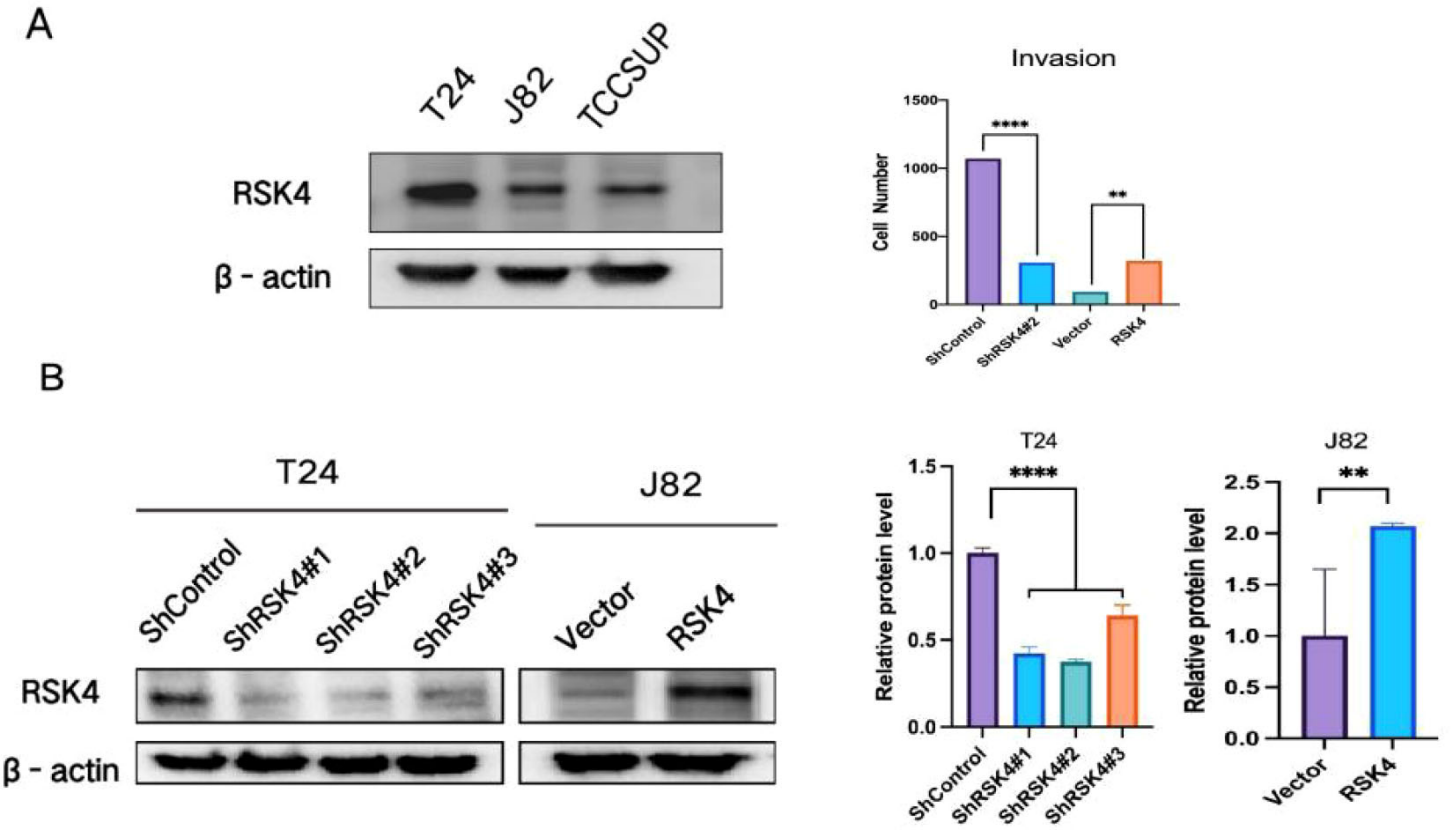
Expression of RSK4 in BLCA cell lines. **(A)** Expression of RSK4 in BLCA cell lines. **(B)** Successful establishment of BLCA cell lines with overexpression of RSK4 (hRSK4) and knockdown of RSK4 (shRSK4) using lentiviruses. Data are presented as mean ± SEM. **p < 0.01, ****p < 0.0001 by t-test.

The baseline expression of RSK4 was initially assessed in a panel of BLCA cell lines (**Figure 5A**). Based on this profile, J82 cells were selected for the establishment of a stable RSK4-overexpressing model via transfection with the pcDNA3.1/neo-RSK4 plasmid. In parallel, T24 cells were transduced with lentiviral particles carrying shRNA targeting RSK4 (shRSK4) to generate a stable knockdown model. Control cell lines for each background were established using the corresponding empty vector or a nontargeting shRNA construct. Western blot analysis confirmed the successful manipulation of RSK4 expression in the engineered cells (**Figure 5B**). Consistent with the protein results, the results of the quantitative evaluation revealed that compared with the control cells, the RSK4-overexpressing clones presented significantly elevated protein levels, whereas the shRSK4-transduced clones presented a marked reduction in RSK4 expression.

### RSK4 promotes the metastatic capacity of BLCA cells

**Figure 6:**
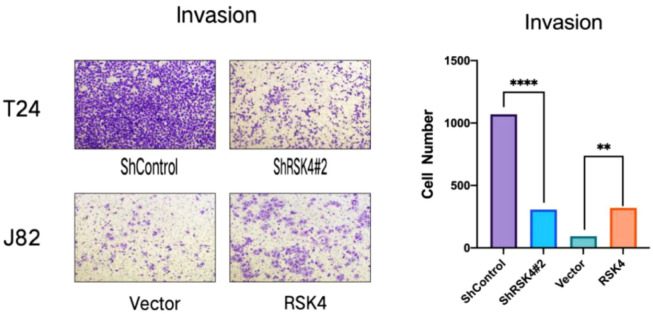
RSK4 expression promotes the invasive capability of bladder cancer cells. Data are presented as mean ± SEM. **p < 0.01, ****p < 0.0001 by t-test.

**Figure 7:**
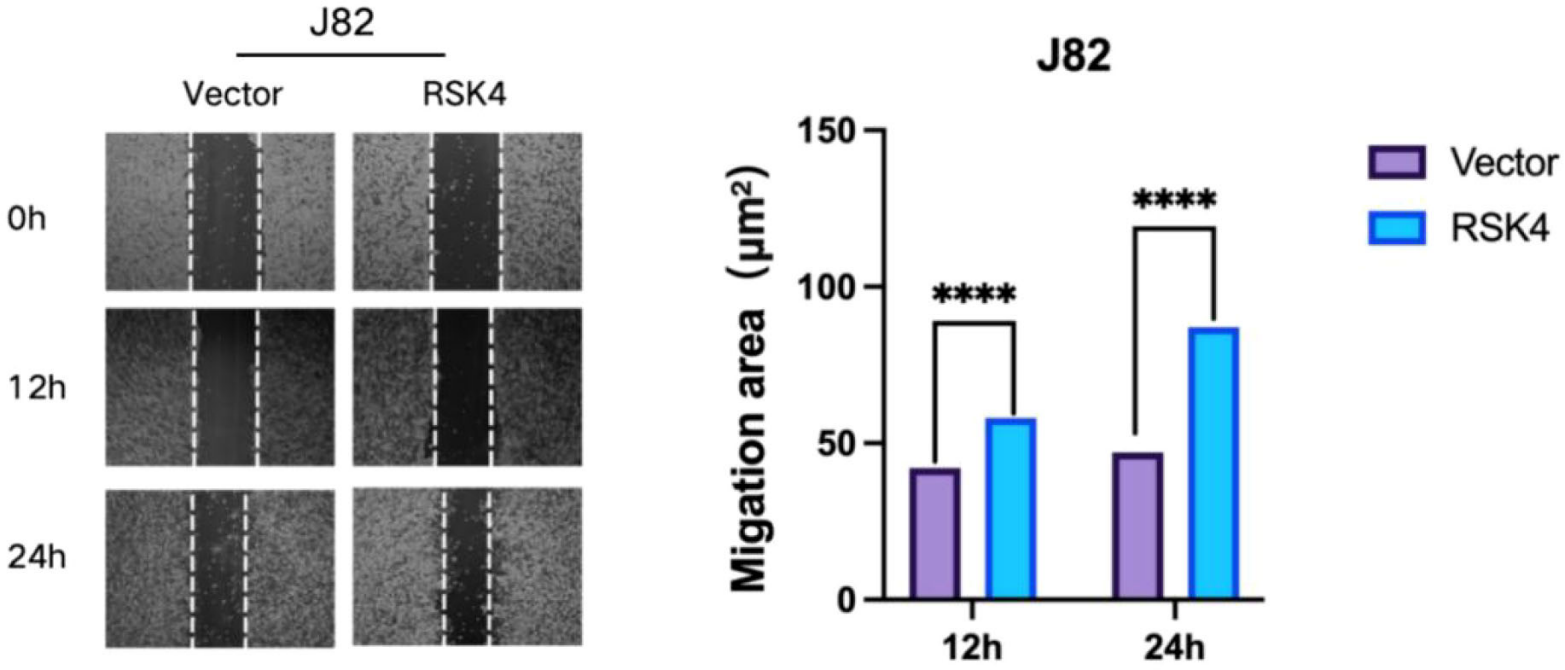
RSK4 expression enhances the migratory capability of bladder cancer cells. Data are presented as mean ± SEM, ****p < 0.0001 by t-test.

To further investigate the impact of RSK4 on the metastatic potential of BLCA, functional assays were performed. Matrigel Transwell invasion assays confirmed that enforced expression of RSK4 promoted cellular invasion, whereas knockdown of RSK4 attenuated this aggressive phenotype (**Figure 6**). In a complementary wound healing assay, RSK4 overexpression significantly enhanced the migratory capacity of BLCA cells (**Figure 7**).

## Discussion

This study demonstrated that RSK4 expression was significantly higher in bladder cancer tissues than in normal tissues (P<0.0001). Positive RSK4 expression correlated positively with tumor muscle layer invasion, high-grade tumors, advanced stages, lymph node involvement, and distant metastasis (P<0.05) but did not significantly differ with age (>60 vs. ≤60 years), sex, or tumor size. Survival analysis revealed lower survival rates in patients with high RSK4 expression, suggesting a potential association with poor prognosis (P<0.05). At the molecular level, RSK4 overexpression enhances the invasive and migratory capabilities of bladder cancer cells. In summary, RSK4 may promote bladder cancer progression and holds promise as a potential prognostic biomarker and therapeutic target, offering new insights for improving clinical outcomes in patients with bladder cancer.

The adverse prognostic factors for bladder cancer are complex and diverse and are primarily determined by the tumor’s clinical stage, pathological characteristics, and molecular biological behavior. First, the most clearly established adverse prognostic factors are tumor stage and grade. Following transurethral resection of bladder cancer, the risks of upper urinary tract recurrence for T1 and T2 stages are 2.5 and 6 times greater, respectively, than that for Ta stage, whereas high-grade (G3) tumors, owing to their increased malignancy, are associated with significantly increased risks of progression and metastasis (12). Second, specific histological subtypes, such as sarcomatoid differentiation (13), micropapillary (14, 15), and plasmacytoid (16) variants of urothelial carcinoma, are typically associated with high invasiveness, resistance to conventional treatments, and poor prognosis. At the molecular level, inactivating mutations in tumor suppressor genes such as TP53 and RB1 (17, 18) have been demonstrated to be correlated with tumor progression and treatment resistance. In this study, for the first time, we propose RSK4 as a novel adverse prognostic factor for bladder cancer. Previous laboratory studies have confirmed that RSK4 accelerates tumor progression as an oncogenic molecule. In this study, patients with high RSK4 expression had significantly reduced overall survival (P<0.05), validating its clinical prognostic value. Moreover, univariate Cox regression analysis revealed that high RSK4 expression was significantly associated with poor overall survival in patients with bladder cancer (HR = 4.603, P<0.01). However, this significance disappeared in the multivariate Cox analysis incorporating classic prognostic factors such as TNM stage, tumor grade, and age. This phenomenon is not uncommon in biomarker prognostic studies (19). The most plausible explanation is confounding effects: RSK4 expression strongly correlates with known strong prognostic factors (e.g., tumor stage), and its significance in univariate analysis partially “represents” the influence of these stronger factors. After adjusting for them in the multivariate model, RSK4’s independent prognostic information becomes relatively weak (20). Similar findings have been reported in other studies (21, 22). Furthermore, univariate analysis suggested that lymph node metastasis and distant metastasis were closely associated with patient prognosis. However, in the Cox multivariate regression model, neither lymph node metastasis nor distant metastasis demonstrated independent prognostic value. We attribute this primarily to the limited sample size and relatively insufficient event numbers in this study, which reduced statistical power and masked their independent effects.

These findings suggest that RSK4 may serve as a key oncogenic factor in the initiation and progression of bladder cancer (BLCA). Our observation of a positive correlation between RSK4 overexpression and muscle layer invasion as well as advanced staging in bladder cancer strongly indicates that RSK4 may promote the invasion and progression of bladder cancer. Its potential mechanisms may involve the phosphorylation of downstream substrates by RSK4, thereby influencing epithelial–mesenchymal transition (EMT) and cell cycle processes or inhibiting apoptosis. ARECHAVALETA-VELASCO et al. (3) reported that RSK4 protein expression is significantly upregulated in ovarian tumors and that high levels of RSK4 are closely associated with high tumor grade, advanced stage, and shorter progression-free survival. Furthermore, both cisplatin and paclitaxel rapidly downregulate RSK4 expression, further confirming its pivotal role as an oncogenic factor in ovarian cancer progression. In esophageal squamous cell carcinoma (ESCC) (8), ΔNp63α drives high RSK4 expression by directly binding its promoter and transcriptionally activating its expression. RSK4 subsequently phosphorylates GSK-3β at Ser9, inhibiting its kinase activity and blocking β-catenin ubiquitination-mediated degradation. This promotes the nuclear accumulation of β-catenin, which forms a transcriptional complex with TCF to activate stemness-related genes such as SOX2, OCT4, and NANOG. This signaling axis enhances the self-renewal and spheroid-forming capacity of CD90^+^/ALDH1^+^ tumor stem cells, significantly amplifying tumor stemness characteristics and driving malignant progression and therapeutic resistance in ESCC. However, RSK4 gene knockdown weakens p53-mediated cell cycle arrest by suppressing p21 expression (23). Lopez-Vicente reported that RSK4 induces cellular senescence by activating the p53–p21 axis. Its expression is widely downregulated in tumors, and RSK4 deficiency allows cells to bypass senescence barriers and continue proliferating (24). These findings suggest that RSK4 may have tumor-suppressing functions. Studies have shown that RSK4 expression is lost in endometrial carcinoma cell lines and primary tumors because of promoter hypermethylation, potentially acting as a tumor suppressor gene involved in tumor progression (6). Furthermore, Thakur et al. (7) reported that high RSK4 expression in breast cancer significantly inhibits metastatic capacity. Its antimetastatic mechanism is mediated by blocking the MAPK/ERK signaling pathway, downregulating the expression of the matrix metalloproteinase MMP-2/9, and inhibiting epithelial–mesenchymal transition (EMT). Our study revealed a clear association between RSK4 expression and clinicopathological phenotypes and further demonstrated that RSK4 enhances the invasive and migratory capacities of bladder cancer cells *in vitro*; however, the underlying molecular mechanisms are still not fully understood. Current evidence suggests that RSK4 may facilitate tumor progression by regulating core cellular phenotypes, including invasion, metastasis, and potentially the cell cycle or apoptosis. Nevertheless, its specific phosphotargets, regulatory kinases, and interactions with pivotal pathways, such as MAPK, PI3K/AKT, and Wnt/β-catenin, await further clarification, thus constituting an important direction for subsequent research.

Current radiotherapy and chemotherapy regimens for bladder cancer remain largely ineffective and offer patients limited long-term survival benefits (25). For instance, platinum-based chemotherapy combined with conventional radiotherapy achieves only a 40–50% objective response rate in patients with advanced bladder cancer, with a median survival extension of less than 4 months (26, 27). Furthermore, the extreme scarcity of therapeutic targets for bladder cancer has long constrained breakthroughs in targeted therapy and its clinical application. Chrysostomou et al. (28) achieved a pivotal breakthrough in this challenging landscape: they first identified RSK4 kinase as a core driver of chemotherapy resistance and tumor metastasis and innovatively proposed a “repositioning of existing drugs” strategy. This involved repositioning marketed fluoroquinolone antibiotics (such as norfloxacin and ciprofloxacin) as highly selective RSK4 inhibitors. *In vitro* experiments and mouse model results consistently demonstrated that these “repositioned fluoroquinolones” effectively reversed cisplatin resistance, inhibited YAP/TAZ-mediated epithelial–mesenchymal transition (EMT), significantly prolonged survival, and reduced distant metastasis. This discovery not only fills the long-standing gap in bladder cancer targeted therapy but also offers a novel pathway to overcome the current clinical objective response rate bottleneck of only 40–50%. Moreover, it establishes a novel combination therapy strategy with rapid clinical translation potential, promising substantial improvements in long-term survival outcomes. Concurrently, our research group has conducted a series of studies on RSK4 small-molecule inhibitors and has filed related patents. Research has indicated that APY29 and CZC-54252 efficiently inhibit RSK4 enzymatic activity, significantly promote apoptosis in esophageal squamous cell carcinoma (ESCC) cells, and effectively block their proliferation, invasion, and migration. These findings not only lay a solid foundation for further clinical translation but also provide a potential therapeutic target for bladder cancer. However, this study has certain limitations. First, this was a single-center retrospective study with a limited sample size, and the conclusions require validation through larger-scale, multicenter prospective studies. Second, the findings of this study confirm the role of RSK4 in promoting bladder cancer progression through both clinical correlations and functional assays (invasion and migration) and preliminarily establish its value as an adverse prognostic factor and potential therapeutic target. However, its specific molecular mechanisms—such as upstream/downstream regulatory networks and precise control of malignant biological behaviors—require systematic validation. Future studies should delve deeper into the specific functions of RSK4 in proliferation, invasion, metastasis, and chemotherapy resistance using cell lines and animal models. These approaches will provide new theoretical foundations and intervention strategies for precision treatment of bladder cancer.

## Data Availability

The original contributions presented in the study are included in the article/supplementary material. Further inquiries can be directed to the corresponding authors.
